# Perspectives of teachers at music schools toward children’s extra-musical abilities promoted by music lessons

**DOI:** 10.3389/fpsyg.2023.1190731

**Published:** 2023-07-10

**Authors:** Satoshi Kawase, Yuriko Kitabayashi

**Affiliations:** ^1^Yamaha Music Foundation, Tokyo, Japan; ^2^The Faculty of Psychology, Kobe Gakuin University, Kobe, Japan

**Keywords:** music lesson, music teacher, non-cognitive skills, teaching experience, teachers’ views

## Abstract

Musical activities foster children’s social ability. However, the question remains whether professional music teachers consider musical training to have an influence on extra-musical abilities or only on the acquisition of musical skills. We conducted an online survey to collect open-ended responses regarding this question from over 2,000 music teachers in one of the biggest extracurricular fee-charging music school groups in Japan. Teachers’ free descriptions were classified into non-cognitive skills, and frequently occurring words were extracted through text mining. The results showed that, although the primary goal of music teachers who provide fee-charging lessons may be to teach musical skills to their students, they were aware of the effects of music lessons on children’s non-cognitive development. Our study’s main findings include: (1) despite free-descriptions, 92% of teachers reported that children acquired extra-musical abilities, and 98% of these responses were categorized as non-cognitive skills, (2) the most common responses within non-cognitive skills were “collaboration,” “task performance,” and “engaging with others”, and (3) there was a significant positive correlation between teaching experience and frequency of mentioning non-cognitive skills. Views on extra-musical abilities, which experienced music teachers especially had, may improve less-experienced teachers’ perspectives on teaching as well as their social roles and occupational awareness.

## 1. Introduction

Children’s music-related abilities can be promoted through musical training. It has been argued that music lessons develop music-related abilities, such as composition, sight-reading skills, and improvisation (McPherson and Gabrielsson, 2002). These skills correspond to the ability to process musical tone in a musical context and process music-related information quickly – essential abilities for proficient performance.

In contrast, it is known that extra-musical abilities, that is, abilities that do not appear to be directly related to musical abilities, are acquired through music lessons. These extra-musical abilities can be understood as cognitive abilities related to the auditory sense. These include the ability related to the discrimination of pitch (Schellenberg and Moreno, 2010), memorizing sounds (Cohen et al., 2011), sensitivity to speech sounds (Magne et al., 2006), and discrimination of emotions from sounds (Thompson et al., 2004). It is presumable that music training also facilitates language processing by promoting abilities related to temporal processing in the brain (Overy, 2003). These findings suggest that musical training affects and possibly improves cognitive abilities that are primarily related to auditory processing.

Meanwhile, recent research suggests that music activities also facilitate non-cognitive extra-musical abilities. These skills are also referred to as socio-emotional skills or personality skills. While cognitive skills are mental abilities such as basic cognitive skills, knowledge acquisition, and reasoning, non-cognitive skills are abilities that affect daily life, such as cooperation with others, emotional control, and perseverance in achieving of goals (Organization for Economic Co-operation and Development, 2015). Previous studies show that non-cognitive skills that can be acquired through musical activities include prosociality (Schellenberg et al., 2015; Williams et al., 2015), sociability (Hallam, 2010; Ilari, 2016; Kawase et al., 2018), empathy (Rabinowitch et al., 2013), helping behavior (Cirelli et al., 2014), and emotional ability (Campayo-Muñoz and Cabedo-Mas, 2017). In particular, collaborative musical activity with others is associated with sociability (Hallam, 2010), and helping behavior for children (Kirschner and Tomasello, 2010). With respect to group music lessons, learning with other pupils can be effective social training for children aged 4–7 years (Kawase et al., 2018).

Musical activities also influence the development of other extra-musical abilities such as socio-emotional factors (Campayo-Muñoz and Cabedo-Mas, 2017) through its influence on emotional competence (Saarikallio, 2019) and emotional regulation (Saarikallio, 2011). Regarding emotional intelligence in music education, children who receive musical education score higher on emotional comprehension tests than those who do not receive such education (Schellenberg and Mankarious, 2012). In other studies, children’s self-esteem (Lillemyr, 1983; Costa-Giomi, 2004; Kokotsaki and Hallam, 2007; Rickard et al., 2013), and self-regulation (Winsler et al., 2011; Brown and Sax, 2013; Williams, 2018) are influenced by music activities.

Nevertheless, it remains unclear how professional music teachers consider extra-musical abilities in their lessons for children. It is noteworthy that although music education involves children, teachers, and parents, perspectives of music teachers who specialized in improving musical skills toward the possible non-cognitive abilities of their pupils have been rarely clarified. This study therefore embarks to examine this. Identifying music teachers’ perceptions of the effectiveness of their teaching is important for determining the beliefs on which music teachers base their teaching practices.

McClung (2000) suggested that a number of music teachers considered that grading in music class should reflect socio-emotional skills as well as music skills. Barrett et al. (2019) conducted a survey in Australia using the Music Beliefs Questionnaire among educators who did not specialize in music. Their results showed that most educators agreed that musical activities were related to children’s social and creative development. However, they also indicated that the aesthetic aspect of music education is highly valued as a benefit of music education and that, even for educators who do not specialize in music, music education tends to focus mainly on creative and aesthetic activities. In light of these findings, it can be argued that, while the acquisition of musical ability is seen as a primary goal, music teachers may also be aware of other abilities and characteristics that are acquired in parallel.

One possible reason for these constructive teachers’ perspectives toward extra-musical abilities is that school education contains different types of curriculums: an explicit one that aims at the acquisition of cognitive skills and another hidden one that aims at the acquisition of social and disciplinary skills throughout school life (McClung, 2000). McClung (2000) also suggested that extra-music socio-emotional competencies have a significant impact on music education, and that they should be integrated into the instructional curriculum.

Meanwhile, the main target of prior studies on extra-musical abilities fostered through music has been the school setting. For music education at school in Japan, where this study was conducted, and in Japan’s Courses of Educational Guidance (set by the Ministry of Education, Culture, Sports, Science and Technology), the goals of music education include acquisition of musical skills and the cultivation of musical ability, as well as the development of emotional sentiments through this ability (Ministry of Education, Culture, Sports, Science and Technology, 2017).

Even so, the perspectives of music teachers who provide extracurricular and fee-charging music lessons for the purpose of improving musical ability remain unclear regarding children’s extra-musical abilities. The primary goal of music schools is the development of musical ability. Indeed, grade examinations to measure musical skills (e.g., immediate reproduction and playing the melody [sound] one listens to, sight-reading, playing accompaniment, and improvisation) exist in the schools targeted by the present study, and systematic technical instruction is provided accordingly.

Compared to the time of the teacher teaching in kindergartens and elementary schools with the students, the time of teacher with the students is as short as a lesson (typically a few dozen minutes per week) in music classes held as extracurricular activities. Therefore, collecting extensive reports from such music teachers would not only reveal their beliefs regarding the extra-musical abilities that children should aim to develop in situations where the transmission and acquisition of music skills are primary, but would also provide indications as to how the music lessons alone are affecting the children. Moreover, this survey’s findings may contribute to more in-depth experimental research.

Regarding potential extra-musical abilities that teachers might be conscious of, this study especially focused on non-cognitive skills that have been regarded as important for life skills throughout a long-life span (Heckman and Rubinstein, 2001; Heckman et al., 2006; Kautz et al., 2014). Because non-cognitive abilities encompass a wide range of abilities that are developed by musical activities, as the aforementioned studies suggest, such skills are expected to have a synergistic effect on the child’s future social and economic success and their cognitive abilities. For example, non-cognitive abilities have been reported to be associated with increasing children’s access to higher education, maintaining good mental health, and future cognitive skills and wellbeing (Organization for Economic Co-operation and Development, 2015).

The categorization of non-cognitive skills used in the present study includes five skills (i.e., engaging with others, collaboration, emotion regulation, task performance, and open-mindedness; see John and De Fruyt, 2015). This categorization was based on a five-factor model of personality (McCrae and Costa, 1996). Cooperative skills or emotion regulation have been found to be related to musical activities (e.g., Hallam, 2010). Likewise, the “task performance” related to non-cognitive skills must also be necessary for musical performance, such as steady practice (Lehmann, 1997) or self-regulation (McPherson and Renwick, 2011).

To address the identified research gaps and questions, we conducted an online survey with over 2,000 music teachers in Yamaha Music Schools that are the largest nationwide system of music schools in Japan and were developed by Yamaha Music Foundation. In 2021, there were approximately 311,000 students enrolled in such schools across Japan and 156,000 students overseas. A total of 18,600 teachers were registered with Yamaha Music Schools in 2021. Children, from infants to high school students, are the main target population of Yamaha Music Schools. The main courses provided comprise modest-sized group lessons. The philosophy of the Yamaha Music School are: “To develop the musical sense that all people possess, and to develop their skills to create music themselves, perform, and enjoy music, in order to spread the joy of music” (Yamaha Music Foundation, 2016). Students can evaluate their own skill level by taking graded exams.

To cover the broader perspective of music teachers in music schools that specialize in the acquisition of musical skills, we collected open-ended responses on the extra-musical abilities that teachers felt children should acquire. The use of the same systematic teaching method would allow us to compare many teachers’ views under similar conditions.

By targeting a group of teachers who use a shared and systematic teaching method, we can also examine the influence of teachers’ experiences on their teaching views, independent of individual teaching methods (e.g., Bautista et al., 2017; Barrett et al., 2019). This expectation is based on the finding that teachers learn through teaching experience (Schmidt, 2010), and that experienced teachers and novice teachers teach using different methods (Hogan et al., 2003). The study’s research questions are as follows:

**RQ1.** Do music teachers consider extra-musical abilities as being capable of being developed through music lessons?

**RQ2.** What types of extra-musical abilities, that is, cognitive or non-cognitive abilities, do music teachers think children acquire?

**RQ3.** Are music teachers’ perspectives on extra-musical abilities related to teaching experience?

## 2. Methods

### 2.1. Participants

Music teachers in Yamaha Music Schools across Japan participated in our online survey. The survey was sent to 6,184 teachers and 2,187 responded. Among them, valid survey responses were obtained from 2,032 teachers. Accordingly, a substantial number of participants from the same music school group reflects the large proportion of music teachers teaching there. All survey responses were from female teachers who were providing group music lessons for preschoolers and elementary school students aged 4 to 6 at Yamaha Music Schools for at least 2 years. The reason for including only female teachers is because, in Japan, there is an overall trend of music schools having more female teachers.

### 2.2. Materials

The questionnaire used in this study contained items on the frequency of teaching preschoolers (rarely/once every few years/about every other year/one class almost every year/multiple classes almost every year) and the number of students to whom teachers have taught lessons for more than 2 years (less than 50 students/about 50–100 students/about 100–300 students/about 300–500 students/about 500–700 students/more than 700 students). After responding to these items, teachers were asked to describe whether they considered their students to have acquired abilities for daily life, other than musical skills such as the ability to dictate and perform music and read scores through musical lessons. If teachers responded “yes,” they were asked to describe the details in an open-ended space. A separate space was provided for information regarding preschoolers (aged 4–6 years) and elementary school students (aged above 7 years old).

### 2.3. Procedure

The survey was conducted online. A link to the survey was sent to teachers registered with Yamaha Music Schools. Teachers were familiar with online surveys, having routinely received a variety of guides and questionnaires. Data were collected from participants who consented to participate in the study. The informed consent form provided to the participants declared that the anonymity of the participants would be ensured, that they would not be advantaged/disadvantaged for not responding, and that the data would not be used for any other purpose other than for the present study. The survey was conducted according to the code of ethics of the Japanese Psychological Association. The average response time was approximately 15 min.

### 2.4. Analysis

We set eight categories for the classification of the responses. These response categories included five categories of non-cognitive skills (i.e., engaging with others, collaboration, emotion regulation, task performance, and open-mindedness; see John and De Fruyt, 2015), two categories that were frequent responses (i.e., listening to others, cognitive skills that included literacy, academic ability, and memory) but could not be classified into non-cognitive skills, and a final “other” category that was not relevant to the five non-cognitive skills or the aforementioned two categories.

This study followed the non-cognitive skill categorization of John and De Fruyt (2015) as it covers a wide range of non-cognitive abilities. In addition, each category is compatible with the five-factor personality model (McCrae and Costa, 1996), respectively. That is, the above five factors are equivalent to extraversion, agreeableness, emotional stability, conscientiousness, and openness to experience. This five-factor model has already been used in research and includes a variety of characteristics, such as empathy and sociability that have been verified as influences of music lessons in previous studies (Chernyshenko et al., 2018). To capture a large number of responses—rarely as short as one word—broadly and without omissions, we used an existing model of non-cognitive skills that encompasses a wide range of concepts. This enabled us to perceive trends in the data regarding what many teachers observed in their students.

The open-ended responses thus obtained were categorized by three judges who assisted the assignment of responses to categories into the above five non-cognitive categories (i.e., “engaging with others,” “collaboration,” “emotion regulation,” “task performance,” and “open-mindedness”) and the other three aforementioned categories (i.e., “listening to others,” “cognitive skills,” and “others”). In total, there were eight categories. If phrases in the teachers’ responses contained more than one characteristic, each factor was categorized separately. For example, if the participants’ free descriptions contained the words “collaboration” and “never give up,” these were classified as collaboration and task performance, respectively. If the judges considered classification difficult, the judges and the authors discussed sorts and shared the information. The mean for all categories of the 36-response Fleiss’ kappa, independently categorized by three judges, was 0.58 (SD = 0.25).

Finally, to scrutinize the contents of participants’ responses and pinpoint common tendencies, we compiled the most frequently occurring words using the text mining software KH coder (Higuchi, 2016). Consequently, we can elucidate teachers’ common views from their free descriptions.

### 2.5. Contents of group lessons that teachers provided in the present study

In Yamaha Music Schools, children and parents, except for elementary school students (described below), participated in a lesson together, which was conducted by a teacher. The age at which children began their lessons differed among groups: from around 1 year-old, 2 years-old, 3 years-old, 4–5 years-old, or 6 years-old (or first grade). In the lessons for each age group, teachers often used a piano, an electronic organ (Electone), or percussion.

The present study targeted teachers who were providing lessons for children aged over 4 years in Yamaha Music Schools. For children aged between 4–6 years, parent–child pairs participate in group lessons 40 times a year (i.e., 3–4 times per month) for 60 min per session. The maximum capacity of the lessons was ten parent–child pairs (usually 4–6 parent–child pairs attended on average). Each pair sat with access to an electronic organ. The content of the lessons was as follows: comprehensive musical study using repertoire pieces, integrated study using musical pieces in the repertoire (listening, sing, playing, and reading), solfeggio, playing easy accompaniment music, playing in an ensemble, and so on. The main instrument used in the lessons was an electronic organ or piano. Regarding elementary school students, only children (i.e., without parents) participated in the group lessons, whose style and duration of study were similar to the lessons for preschoolers, which were conducted three times per month. The lessons include the study of harmony, ensemble performance, arrangement singing, reading musical notation, musical grammar, and playing solo repertoire pieces.

## 3. Results

Of the participants, 91.6% reported that children acquired extra-musical abilities. The number of years of teaching experience of the participants who answered that children could acquire extra-musical ability was significantly higher than that of the participants who did not, *M*_yes_ = 24.3 years, *M*_no_ = 21.3 years, *t* (146.97) = 3.22, *p* = 0.002.

**Figure 1:**
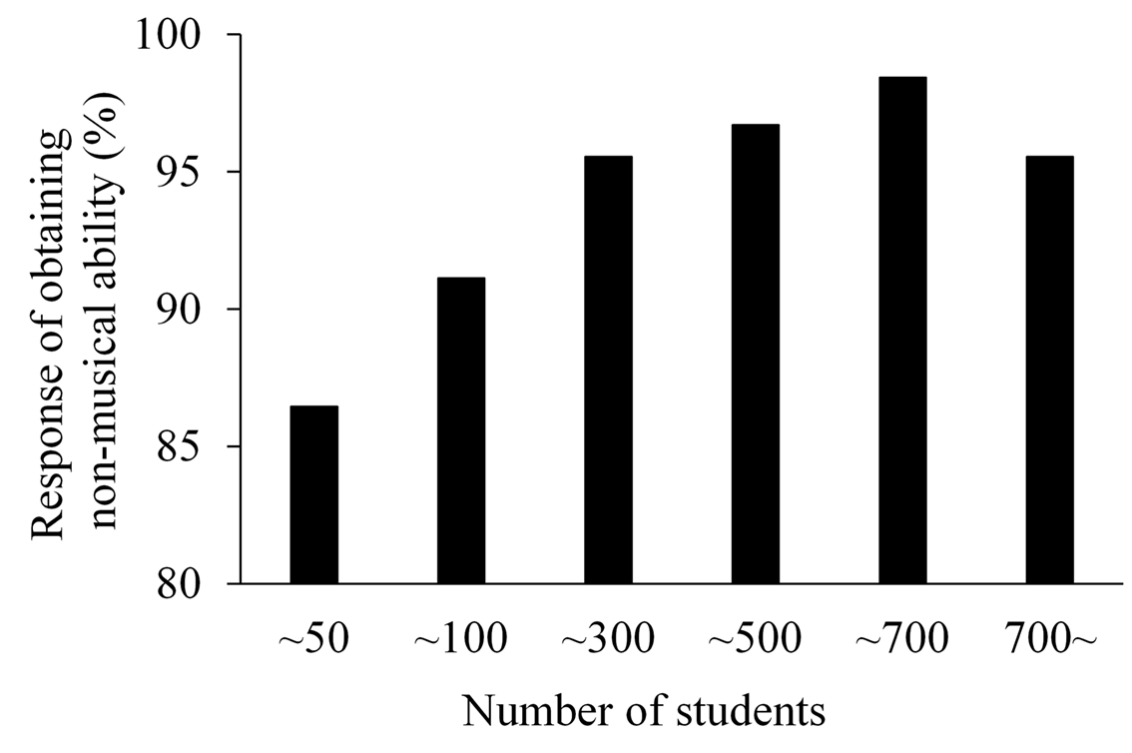
The number of students to whom teachers provided lessons for 2  years or more. The horizontal axis represents the number of students and the vertical axis represents the proportion of teachers who responded that children have acquired extra-musical abilities.

Figure 1 shows the number of students who received lessons for more than 2 years and the percentage of teachers who responded that the children obtained extra-musical abilities. As a general tendency, music teachers who provided lessons to a larger number of students were more likely to answer that children acquired abilities other than strictly musical ones. The results of a chi-square test showed that the percentage of responses that indicated that children obtained extra-musical abilities increased as the number of children who were provided lessons increased, *χ*^2^(5) = 48.19, *p* < 0.01. Teachers who provided lessons for fewer than 50 students and teachers who provided lessons for fewer than 100 students showed a significantly lower proportion of “yes” responses (*p* < 0.05) than teachers who provided lessons for a larger number of students. However, there was no significant difference regarding proportion of “yes” responses between teachers who provided lessons for less than 100 students and those who provided lessons for more than 700 students. There was also no significant difference among teachers who provided lessons for more than 300 students.

**Figure 2:**
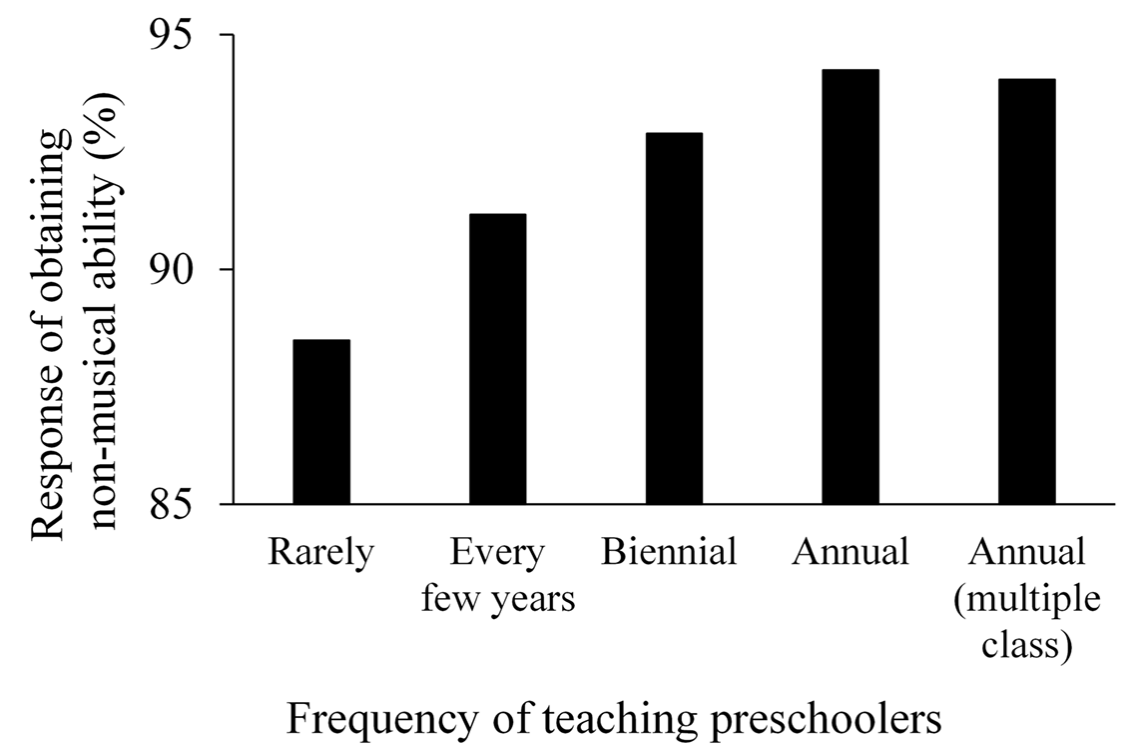
The frequency of lessons for preschoolers. The horizontal axis represents the frequency of lessons and the vertical axis represents the percentage of teachers who answered that children have acquired abilities other than musical ones.

A chi-square test was conducted to examine the relationship between frequency of teaching preschoolers and ratio of teachers who reported that their students acquired extra-musical abilities (Figure 2). The results showed that teachers rarely assigned to teach preschoolers were less likely to respond that children acquired extra-musical abilities, *χ*^2^(4) = 8.48, *p* = 0.08. The percentage of teachers who reported that children acquired extra-musical abilities was significantly higher (*p* < 0.05) among teachers who had been assigned to one or more classes every year than among teachers who had been assigned to few lessons for preschoolers.

Even though teachers were free to describe specific aspects of children’s non-musical abilities fostered by music lessons, 97.9% of the total completed responses to open-ended questions about extra-musical abilities involved single or several non-cognitive skills, while cognitive skills were not mentioned as much. The number of open-ended responses regarding lessons for preschoolers was 1,705, and regarding lessons for elementary school students, the number was 1,570. These numbers represent teachers who wrote answers in the open-ended responses. Some teachers noted nothing in the open-ended responses, whereas others reported in the open-ended response for both (preschoolers and elementary school students).

**Figure 3:**
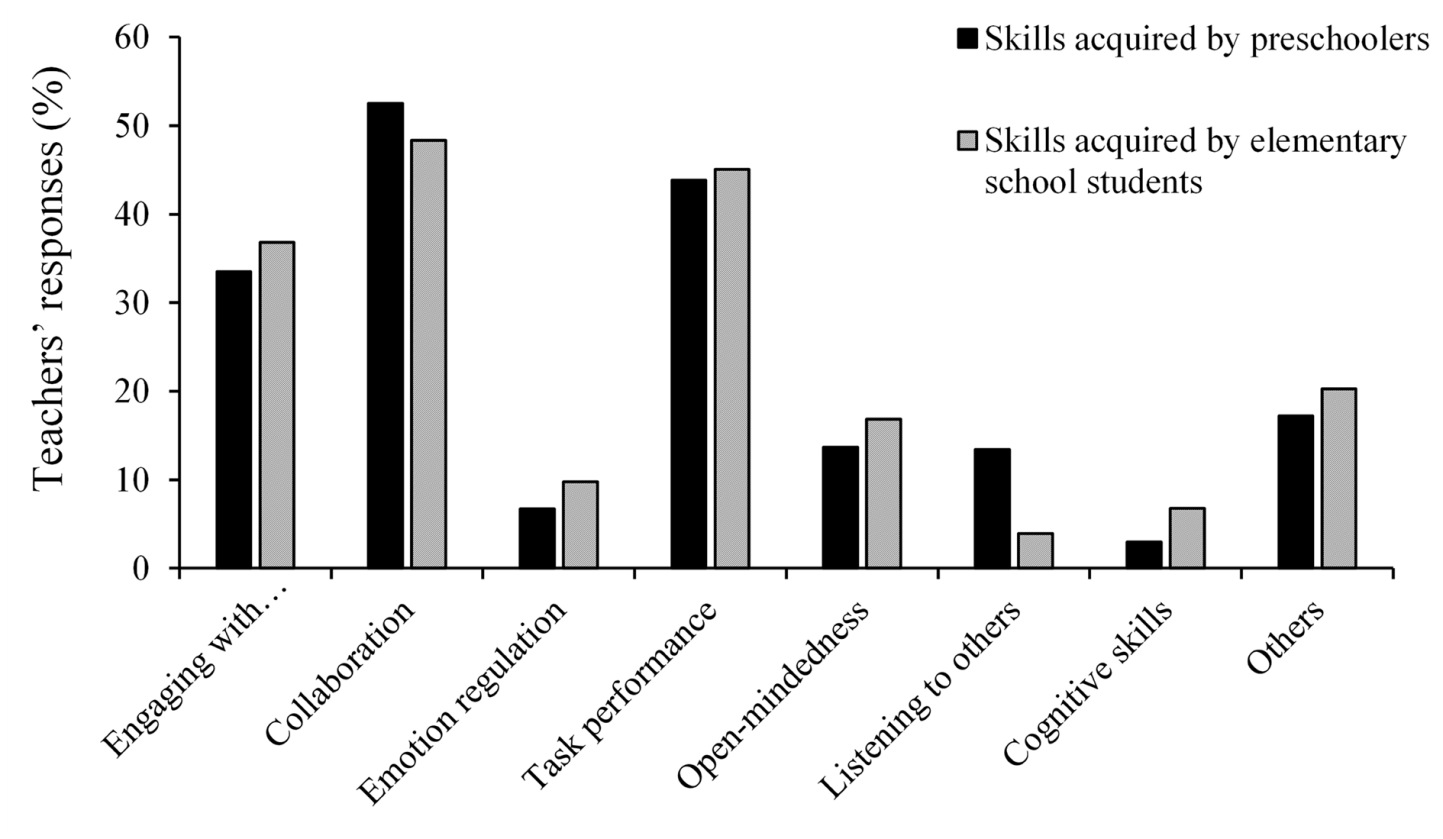
The percentage of responses pertaining to each category included in the open-ended responses. The vertical axis represents the percentage responses pertaining to each category out of all completed responses, and the horizontal axis represents each category.

Figure 3 represents the percentage of teachers’ responses related to the non-cognitive skills acquired by preschoolers and elementary school students, classified by category, that is, the five categories of non-cognitive skills (i.e., engaging with others, collaboration, emotion regulation, task performance, and open-mindedness), one category of listening to others, one category of cognitive skills, and one category related to other skills. Regarding the non-cognitive skills acquired by both preschoolers and elementary school students, the most common responses provided by the teachers were collaboration, task performance, and engaging with others. Therefore, our results indicated that the participants considered these three non-cognitive skills as the main extra-musical abilities acquired by children through music lessons.

**Figure 4:**
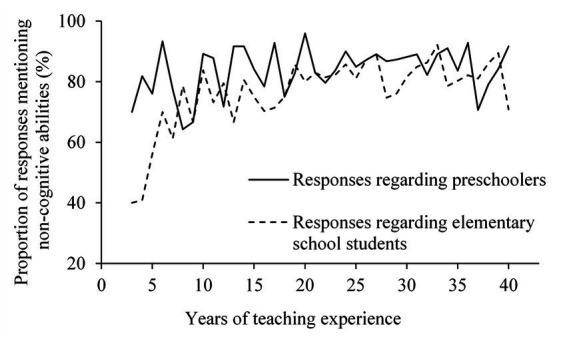
The relationship between mentions of non-cognitive abilities and teaching experience. The vertical axis represents the percentage of teachers who mentioned non-cognitive skills from among those who answered that extra-musical abilities would be acquired by children. The horizontal axis represents the number of years of teaching experience.

We examined the relationship between references to non-cognitive skills and years of teaching experience. Figure 4 shows the relationship between the mention of any of the five non-cognitive skills and teachers’ years of teaching experience. For responses regarding preschoolers, many teachers mentioned non-cognitive skills even when they had fewer years of teaching experience, while for responses regarding elementary school students, mentions of non-cognitive skills increased with the number of years of teaching experience. The correlation coefficients between teachers’ years of teaching experience and mention of non-cognitive skills were significant for both preschoolers and elementary school students (*r*_preschoolers_ = 0.33, *p* = 0.05; *r*_elementary school students_ = 0.65, *p* < 0.01).

The relationship between the categories of content mentioned by teachers in their open-ended responses and their years of teaching experience were calculated (Table 1). The results showed that regardless of the students’ age, as the number of years of teaching experience increased, so did the references to “engaging with others,” “emotion regulation,” and “task performance.” “Collaboration” was frequently mentioned in responses regarding elementary school students. Furthermore, “open-mindedness” and “cognitive skills” were mentioned more in responses pertaining to preschoolers as the number of years of teaching experience increased.

**Table 1:** Correlation coefficients between years of teaching experience and percentages mentioned in each category.

	Preschoolers	Elementary school students
Engaging with others	0.36^*^	0.56^**^
Collaboration	0.26	0.47^**^
Emotion regulation	0.27†	0.46^**^
Task performance	0.33^*^	0.70^**^
Open-mindedness	0.33^*^	−0.14
Listening to others	−0.32^*^	0.17
Cognitive skills	0.51^**^	0.25

^**^p < 0.01, ^*^p < 0.05, and ^†^*p* < 0.1.

Table 2 summarizes the top 10 words obtained through text mining. Here, adjunctive words and phrases (e.g., “ability of …” or “I think …”) are omitted. The most frequently occurring words were “collaboration,” “concentration,” and “friends” (Table 2). These words are specific examples of teachers’ views on non-cognitive skills. “Friends” included being able to get along with friends, being considerate of friends, and being able to respect friends. As Table 2 shows, many of the words and phrases in the teachers’ responses were related to “others,” such as “collaboration with others,” “others and self,” and “self-expression.” In addition to these words related to non-cognitive abilities, the word “school” was also used to describe having friends outside of elementary schools.

**Table 2:** Most mentioned words in teachers’ responses.

Preschoolers		Elementary school students	
Word	Ratio	Word	Ratio
Collaboration	25.5	Collaboration	15.5
Concentration	24.4	Friends	14.4
Friends	17.4	Oneself	14.1
Conversation	11.3	Concentration	11.7
Listen	10.6	Effort	9.7
Social	9.8	School	8.2
Effort	8.3	Music	7.8
Lesson	7.7	Thinking	7.6
Oneself	7.7	Consideration	7.3
Capability	7.0	Feelings	7.2
			(%)

## 4. Discussion

This study aimed to reveal professional music teachers’ views regarding the impact of music education on children’s acquisition of extra-musical abilities. Our large-scale data clarified that although the primary goal of music teachers who provide fee-charging lessons at music schools may be to impart musical skills to their students, music teachers were aware of the effects of music lessons on children’s non-cognitive development. The main findings that are relevant to our RQs were as follows: (1) Almost all music teachers considered that musical lessons fostered and had a positive impact on extra-musical abilities. Even though music teachers were free to describe extra-musical abilities as they pleased, the number of mentions of cognitive skills was lower than that of non-cognitive skills, (2) Among non-cognitive skills, collaboration, task performance, and engaging with others were the ones most mentioned by the music teachers, and (3) The more experience music teachers accumulated, the more they believed that children acquired extra-musical abilities.

### 4.1. High awareness of extra-musical abilities fostered by music lessons

Despite being allowed to provide free-descriptions, 92% of the music teachers pointed out that music lessons fostered extra-musical abilities. Most of their responses contained content concerning non-cognitive skills as opposed to cognitive skills. This suggests that almost all of the teachers were aware that musical lessons for children affected children’s development of non-cognitive skills, although one of the primary goals of music teachers providing fee-charging lessons may be “for musical skill acquisition” (and participants in this study were extracurricular professional music teachers who taught only within a limited duration and did not teach other subjects). While McClung’s (2000) study indicated that some music teachers only emphasized musical skills, it was notable that almost all the teachers in this study emphasized extra-musical abilities. In addition, this result contrasts with that suggesting that aesthetic aspects of music education are highly valued as a benefit of music education (Barrett et al., 2019).

Furthermore, in light of the findings that teachers’ beliefs can significantly influence children (Kunter et al., 2013), the present results suggest that children’s non-cognitive extra-musical abilities, which are reported to be important for lifelong child development (Heckman and Rubinstein, 2001; Heckman et al., 2006; Kautz et al., 2014) can be extended by such music teachers’ views of non-cognitive skills. At the same time, the present results support previous findings that musical education or activities affect the social abilities of children (Hallam, 2010; Rabinowitch et al., 2013; Williams et al., 2015; Ilari, 2016; Kawase et al., 2018).

It was also notable that even though music teachers were free to describe extra-musical abilities as they pleased, the number of mentions of cognitive skills was lower than that of non-cognitive skills. While many past studies have already indicated the impact of musical lessons on academic skills (Hallam, 2010) or intelligence (Schellenberg, 2011), our results indicated that music teachers pay more attention to the additional effects of musical lessons on non-cognitive skills than just toward cognitive skills. Fewer mentions of cognitive abilities induced by music lessons is partly in accordance with the fact that there are fewer types of cognitive benefits of musical activities when compared to musical and extra-musical activities (Mehr et al., 2013). Thus, the present results on music teachers’ views contribute to contrary findings on prominent or non-prominent effects of music education on cognitive/non-cognitive abilities.

### 4.2. Non-cognitive abilities mentioned by music teachers

Music teachers focused on three primary aspects of non-cognitive skills: collaboration, task performance, and engaging with others. Comparisons between the number of words included in each of the five categories (engaging with others, collaboration, emotion regulation, task performance, and open-mindedness) implied that skills for action with others and fulfillment of tasks were regarded as important.

In particular, the commonly reported words were frequently related to social and interpersonal factors. The results of the text mining showed that the most frequently mentioned was collaboration. “Friends,” which was the frequently mentioned word, obtained the third highest number for preschoolers and the second highest number for elementary school students. One characteristic of these results is that music teachers thought that children acquired non-cognitive skills relevant to the situations where children interacted with each other. This can be explained by the feature of group lessons provided by music teachers in the present study that require children’s attention toward the existence of “many other people,” namely, a teacher, peers, and parents (Kawase et al., 2018).

Given that such musical interactions could enhance children’s prosociality (Kirschner and Tomasello, 2010; Schellenberg et al., 2015; Williams et al., 2015), empathy (Rabinowitch et al., 2013; Kawase, 2016), and sociability (Kawase, 2015; Kawase et al., 2018), teachers were also conscious of such collaborative skills fostered through musical lessons. In addition, music teachers’ views of social and interpersonal factors can be explained by the effects of both collaborative music activities and interpersonal interactions (Keller, 2014; Kawase, 2014a, 2014b; Trehub et al., 2015) or entrainment (Clayton et al., 2020).

Words categorized as “task performance” also occurred frequently. Music teachers’ views were focused on concentration, effort, proactivity, perseverance, confidence, continuity, goals, hard work, etc. These words may reflect the essential ability of children to practice music consistently and achieve excellent music performances, as musical activity requires such actions or mindsets. Considering the finding that steady practice is necessary for proficiency in musical skills (e.g., Lehmann, 1997), it may be natural that skills related to task performance were regarded as notable by teachers. This salient “task performance” is also in accordance with self-regulation enhanced through music activities (McPherson and Renwick, 2011; Winsler et al., 2011; Brown and Sax, 2013; Williams, 2018) and with self-management and discipline influenced by art education that includes music (Farrington et al., 2019).

Contrary to our expectations, the number of mentions of “emotion regulation” was relatively low, although several teachers highlighted these aspects. Based on emotion regulation (Saarikallio, 2011) and self-esteem influenced by music activities (Lillemyr, 1983; Costa-Giomi, 2004; Kokotsaki and Hallam, 2007; Rickard et al., 2013), we expected that emotional aspects would be highlighted by music teachers; however, this was not the case. The reason for this is unclear. However, we can reason that this may be because this study was conducted in an environment largely featuring group lessons that required social interactions for coordination with others, and therefore, the terms “collaboration” and “engaging with others” were more salient for music teachers than emotion regulation. Moreover, since children who originally had a certain degree of emotion regulation might be able to participate in lessons continuously, the growth of such ability might be less noticeable in the present settings.

Despite fewer mentions of “openness,” music teachers’ views of this can be explained by the relationships between music training for children or adolescents and higher openness-to-experience (Corrigall et al., 2013; Hille and Schupp, 2015). Indeed, openness was reported to be a characteristic personality trait in musicians (Corrigall et al., 2013). However, in this study, since music teachers were asked about children’s ability to acquire it in their “daily lives,” it might have been difficult for them to mention creativity and aesthetic interest, which are related to music. Similar to the aforementioned emotion regulation, in group music lessons, aspects such as relationships with others and working diligently may be more noticeable than openness.

### 4.3. Links between teaching experience and perspectives toward extra-musical abilities

Teaching experience could influence the degree of music teachers’ awareness of children’s non-cognitive skills acquired through musical lessons. The present study showed that as music teachers’ educational experience progresses, they become more aware of extra-musical abilities unrelated to their primary objective. In terms of relationships between teachers’ experiences and their subjective views, the results also contribute to the research of non-cognitive skills. In addition, the present results support the idea that music teachers’ experiences could potentially influence their views (Bautista et al., 2017).

These results can be explained by several factors. For example, a longer teaching experience may allow music teachers to pay more attention to aspects other than teaching musical skills. In the early stage of a music teacher’s career, they are likely to focus imparting musical skills, which is the main objective of music lessons. As teachers become more proficient in teaching, it can be inferred that they allocate wider mental capacities to focus on other aspects of music education. This may be a possible reason why it is more difficult to pay attention to non-cognitive abilities in the early stages of music teachers’ careers than in later stages.

Otherwise, it can be presumed that the longer the teachers’ accumulated experience, the more opportunities they have to identify changes in children. In other words, since it is possible that inexperienced music teachers did not, or rarely, encounter situations in which children acquired extra-musical abilities, inexperienced music teachers did not recall such situations in this survey. Moreover, teaching experience can provide teachers with insight (Schmidt, 2010); therefore, teaching experience can broaden music teachers’ perspectives, or improve their holistic teaching skills in addition to teaching music skills in several ways (Berliner, 2001). Differences in teaching methods between novices and experienced teachers in a variety of school subjects may also partly explain this result (Hogan et al., 2003).

Since teaching experience can change perspectives regarding the efficacy of musical education, views of extra-musical abilities may be applicable to the interactive learning of teaching skills between experienced teachers and novices or students who want to become music teachers (Coenders and Verhoef, 2019).

## 5. Conclusion

Our data on professional music teachers’ free descriptions provide evidence of what music teachers in music schools consider as extra-musical abilities acquired through lessons, even though the primary goal of music schools might be the provision of musical skills. Teaching experience could influence teachers’ awareness of children’s non-cognitive skills, which may mean that a teacher’s professional growth is related to the acquisition of a wider perspective on the skills acquired through music lessons. The possibilities of music education for holistic human development can influence teachers’ teaching attitudes and identities. Such impacts may improve not only children’s abilities but also music teachers’ social roles and occupational awareness. Furthermore, in terms of their application to music education, if skills such as those mentioned in this study are fostered, our results may contribute to the development of a rubric for teachers to measure not only musical ability but also various other types of extra-musical abilities of children.

The limitations of the present study are related to the teaching method. This study was conducted with music teachers who provided group lessons outside school. In such a collaborative but limited and short-term situation, there would be more focus on collective activities with others rather than other types of activities, such as those pertaining to the development of emotion regulation or openness. Therefore, it is conceivable that these other aspects may be emphasized in situations such as one-on-one lessons and music lessons in public or private schools. In addition, this study was conducted with out-of-school and music-specific teachers in a professional educational setting. Therefore, additional studies will be necessary to determine whether our results apply to music teachers who interact with pupils in more diverse ways outside of music classes. This could include, for instance, teachers who engage in music education at elementary, middle, and high schools.

Future studies should also investigate long-term changes in children. Non-cognitive skills have been found to be associated with children’s development and future success (Heckman and Rubinstein, 2001; Heckman et al., 2006; Kautz et al., 2014). The present results showed similar tendency in teachers’ views toward preschoolers and elementary school students, although this study was broadly divided into two categories, i.e., preschoolers and elementary school students. Thus, it would be necessary to explore at what age the behaviors related to non-cognitive abilities, as reported in this study, begin to be observed. It may be possible to develop age-appropriate programs for the development of non-cognitive skills by correlating such findings to the educational programs offered to children at each learning and developmental stage. Furthermore, the size of the teaching group would also be considered since smaller lessons would be more likely to provide more attention to each child.

Cultural differences in teachers’ perspectives should also be explored. For example, in this study, the categories of openness and emotion regulation received fewer responses than the other three categories. However, we need to examine whether this tendency is universal in other countries where teachers have different beliefs about music education.

This study also provided suggestions to examine the wide range of possibilities in music education, and clues to solving questions such as: what is it about the lesson that leads to different perspectives of teachers on extra-musical abilities, and is it actually linked to the child’s acquisition of abilities? Do such teachers’ perspectives affect teachers’ behaviors in their lessons? Do such teachers’ perspectives lead to teachers’ self-efficacy and motivation? Such questions for future research will contribute to the comprehension of music lessons not only as a means of acquiring musical skills, but also as opportunities for the holistic development of children and teachers.

## Data availability statement

The raw data supporting the conclusions of this article will be made available by the authors, without undue reservation.

## Ethics statement

Ethical review and approval was not required for the study on human participants in accordance with the local legislation and institutional requirements. The patients/participants provided their written informed consent to participate in this study.

## Author contributions

SK and YK designed the study. SK analyzed and interpreted the data and drafted the manuscript. YK conducted the survey. All authors contributed to the article and approved the submitted version.
